# Covariance-Based Direction-of-Arrival Estimation of Wideband Coherent Chirp Signals via Sparse Representation

**DOI:** 10.3390/s130911490

**Published:** 2013-08-29

**Authors:** Zhichao Sha, Zhengmeng Liu, Zhitao Huang, Yiyu Zhou

**Affiliations:** College of Electronic Science and Engineering, National University of Defense Technology, Changsha 410073, China; E-Mails: liuzhangmeng@nudt.edu.cn (Z.L.); taldcn@gmail.com (Z.H.); zhouyiyu@sohu.com (Y.Z.)

**Keywords:** direction-of-arrival (DOA) estimation, wideband chirp signal, sparse representation, convex optimization

## Abstract

This paper addresses the problem of direction-of-arrival (DOA) estimation of multiple wideband coherent chirp signals, and a new method is proposed. The new method is based on signal component analysis of the array output covariance, instead of the complicated time-frequency analysis used in previous literatures, and thus is more compact and effectively avoids possible signal energy loss during the hyper-processes. Moreover, the *a priori* information of signal number is no longer a necessity for DOA estimation in the new method. Simulation results demonstrate the performance superiority of the new method over previous ones.

## 1. Introduction

Previous direction-of-arrival (DOA) estimation methods for wideband chirp signals are mostly based on the special time-frequency distribution of such signals. Ma and Goh separate the simultaneous chirp signals first according to their distinguishable auto- or cross-terms in the ambiguity function, and then use the secondary time-frequency data to estimate their directions [1]. Their methods are grounded on the assumption that the signals are separable in the time-frequency domain, so they are not usable for completely overlapped coherent chirp signals. Wang and Xia use the chirp rate and coarse DOA estimates to compensate the time-varying array manifold, and then introduce narrowband methods to refine the DOA estimates [2]. This process is repeated several times to obtain converged source directions, but our experiments and previous literature [1,3] show that such expected convergence is not guaranteed, especially when the signal-to-noise ratio (SNR) is low. Gershman and Amin focus the signal energy to a certain manifold in the time-frequency domain, and then use narrowband methods to estimate the directions of wideband chirp signals [3], but as the focusing process may loose some signal energy, the performance of this method at low SNR may deteriorate significantly. A maximum likelihood (ML) method has also proposed by Gershman *et al.* in [4] to address the parameter estimation of polynomial-phase signals generally, but it is computationally too intensive, thus is not suitable for practical applications.

This paper addresses the problem of DOA estimation of multiple wideband coherent chirp signals, which emerges due to various factors such as multi-path and echo signals [5]. The recently interest-attracting technique of sparse representation [6,7] is introduced to solve this problem. To simplify the analysis, we assume in this paper that the central frequency and chirp rate of the coherent signals are known, which is reasonable in cooperative applications or when it has been estimated using other methods. The new method significantly differs from previous ones, as it is based on the array output covariance matrix, and completely avoids time-frequency analysis, thus being much more compact and avoiding possible signal energy losses during the complicated hyper-processes. Furthermore, the new method transforms the problem of DOA estimation of wideband chirp signals to one of sparsely representing an observation vector, thus automatically concentrating the observation data energy on the signal directions, so it does not require the *a priori* information of incident signal number, and it achieves model-order selection simultaneously.

## 2. Problem Formulation

Suppose that the chirp rate of the incident coherent signals is *α*, the starting, ending and central frequencies are *f*_1_, *f*_2_ and *f*_0_, respectively, the chirp period is *T*, signal bandwidth is *B*, which satisfies. *B*= *f*_2-_*f*_1_= *αT*. The temporal waveform of the chirp signal is:

(1) $$\begin{array}{cc} s(t)=\eta \exp\left(j2\pi \left(f_{1}t+\frac{\alpha }{2}t^{2}\right)\right), & 0\leq t\leq T, \end{array}$$

where *η* comprises the signal amplitude and initial phase. When wideband coherent chirp signals impinge onto an *M*-element linear array from directions **θ**= [*θ*_1_,…, *θ_K_*], the array output at time is given by:

(2) x(t)=A(θ,t)s(t)+v(t),

where **s**(t)= [*s*_1_(*t*),…,*s_K_*(*t*)] *^T^* is the signal waveform vector, **v**(*t*) is the additive noise with power, $\sigma_{v}^{2}$, **A**(***θ***,*t*)= [**a**(*θ*_1_,*t*),**a**(*θ_K_*,*t*) ] is the array responding matrix at, *t* which not only depends on the signal directions, but also varies with the instantaneous signal frequency. The time-varying response matrix blocks the straightforward usage of conventional subspace methods in DOA estimation of wideband chirp signals. Existing methods address this problem by exploiting the property of linear frequency modulation of such signals, but they are much too complicated and do not deal with the wideband coherent chirp signals effectively, as they are completely overlapped in the time-frequency domain. This paper aims at the DOA estimation of wideband coherent chirp signals via covariance component analysis, thus avoiding the complicated time-frequency analysis process and possible signal energy loss during this process.

## 3. DOA Estimation of Wideband Coherent Chirp Signals

When *K* wideband coherent chirp signals impinge from directions *θ*_1_,…,*θ_K_* simultaneously, the output of the *m*th sensor at time *t* is given by:

(3) $$x_{m}(t)=\overset{K}{\underset{k=1}{\text{∑}}}S_{k}\left(t-\tau_{k,m}\right)+v_{m}(t)=\overset{K}{\underset{k=1}{\text{∑}}}\eta_{k}\exp\left(j2\pi \left(f_{1}\left(t-\tau_{k,m}\right)+\frac{\alpha }{2}{\left(t-\tau_{k,m}\right)}^{2}\right)\right)+v_{m}(t),$$

where *τ_k,m_* is the propagation delay of the signal from the reference to the *m*th sensor. We take the first sensor as the reference, thus *τ_k_*,_1_ =0. Suppose that *N* snapshots are collected with interval *T_s_*, and the total observation time equals the chirp period of the incident signals, *i.e.*, *T* = *NT_s_*, then the (*p*,*q*) element of the covariance matrix estimate **R̂** is:

(4) $$\begin{array}{cc} {\hat{\text{R}}}_{p,q} & =\text{Corr}\left(x_{p}\left(t\right),x_{q}\left(t\right)\right)=\overset{K}{\underset{k=1}{\text{∑}}}\overset{K}{\underset{k^{\prime }=1}{\text{∑}}}\eta_{k}\eta_{k^{\prime }}^{*}\psi \left(\tau_{k^{\prime },q}-\tau_{k,p}\right)+\varepsilon_{p,q}\\ & \approx \overset{K}{\underset{k=1}{\text{∑}}}\overset{K}{\underset{k^{\prime }=1}{\text{∑}}}\eta_{k}\eta_{k^{\prime }}^{*}\sin\text{c}\left(\pi \alpha T\left(\tau_{k^{\prime },q}-\tau_{k,p}\right)\right)B_{1}+\varepsilon_{p,q},\\ B_{1} & =\exp\left(j\pi \alpha \left(\tau_{k^{\prime },q}^{2}-\tau_{k,p}^{2}\right)\right)\exp\left(j2\pi f_{0}\left(\tau_{k^{\prime },q}-\tau_{k,p}\right)\right)\exp\left(j\pi \alpha T\left(\tau_{k^{\prime },q}-\tau_{k,p}\right)\right) \end{array}$$

where *ψ*(*τ*) is the auto-correlation of the chirp signal with unit amplitude, which satisfies *ψ*(0)=1; the estimation perturbation *ε_p,q_* is:

(5) $${ɛ}_{p,q}=\text{Corr}\left(\overset{K}{\underset{k=1}{\text{∑}}}S_{k}\left(t-\tau_{k,p}\right),v_{q}(t)\right)+\text{Corr}\left(v_{p}(t),\overset{K}{\underset{k=1}{\text{∑}}}S_{k}\left(t-\tau_{k,q}\right)\right)+\text{Corr}\left(v_{p}(t),v_{q}(t)\right)$$

whose expectation is $\text{E}\left({ɛ}_{p,q}\right)=\sigma_{v}^{2}\delta (p-q)$ and satisfies E[*ε_p,q_*(**R̂***_p,q_*−*ε_p,q_*)* ]=0 Further straight-forward derivation shows that the variance of *ε_p,q_* is:

(6) $$\begin{array}{c} \text{Var}\left({\hat{\text{R}}}_{p,q}\right)=\text{E}\left({ɛ}_{p,q}{ɛ}_{p,q}^{*}\right)\\ =\frac{\sigma_{v}^{2}}{N}\overset{K}{\underset{k=1}{\text{∑}}}\overset{K}{\underset{k^{\prime }=1}{\text{∑}}}\eta_{k}\eta_{k^{\prime }}^{*}\left[\psi \left(\tau_{k,p}-\tau_{k^{\prime },p}\right)+\psi \left(\tau_{k,q}-\tau_{k^{\prime },q}\right)\right]+\frac{\sigma_{v}^{4}}{N}\left[1+\delta \left(p-q\right)\right]. \end{array}$$

Equation (4) indicates that when *K* wideband coherent chirp signals impinge simultaneously, each covariance element consists of *K*^2^ “signal” components, including *K* auto-tems and *K*^2^−*K* cross-terms. For large, *K* the structure of the covariance is very complicated, and it is difficult to obtain the signal directions from it. Therefore, we set *q*=1 in Equation (4) to simplify their structure, *i.e.*, we only extract the elements along the first column. The expressions of those elements are:

(7) $$\begin{array}{l} {\hat{\text{R}}}_{p,1}=\left(\overset{k}{\underset{k^{\prime }=1}{\text{∑}}}\eta_{k^{\prime }}^{*}\right)\overset{k}{\underset{k=1}{\text{∑}}}\eta_{k}\sin\text{c}\left(\pi \alpha T\tau_{k,p}\right)B_{2}+\varepsilon_{p,1}.\\ B_{2}=\exp\left(j\pi \alpha \tau_{k,p}^{2}\right)\exp\left(-j2\pi f_{0}\tau_{k,p}\right)\exp\left(-j\pi \alpha T\tau_{k,p}\right) \end{array}$$

As, $\text{E}\left({ɛ}_{1,1}\right)=\sigma_{v}^{2}$, the signal components are contaminated by the unknown noise power in **R̂**_1,1_, thus we extract the 2nd to *M*th elements in the first column to form a new observation vector r̂= [**R̂**_2,1_, …, **R***_M_*_,1_]*^T^*. Define $\eta_{\Sigma }=\overset{K}{\underset{k=1}{\text{∑}}}\eta_{k}$, then **r̂**_1_ can be expressed as follows:

(8) $${\hat{\text{r}}}_{1}=\eta_{\Sigma }^{*}\overset{K}{\underset{k=1}{\text{∑}}}\eta_{k}\psi_{1}\left(\theta_{k}\right)+{ɛ}_{1}=\eta_{\Sigma }^{*}\left[\psi_{1}\left(\theta_{1}\right),\ldots ,\psi_{1}\left(\theta_{k}\right)\right]\left[\begin{array}{c} \eta_{1}\\ \vdots \\ \eta_{K} \end{array}\right]+{ɛ}_{1},$$

where **ε**_1_= [ε_2,1_,…,ε*_M_*_,1_]*^T^*, **ψ**_1_(*θ_k_*)= [{sinc(πα*Tτ_k_*,*_p_*)*B*_2_}*_p_*_=2,…_,*_M_*]*^T^*. **ψ**_1_(*θ_k_*) relies uniquely on the signal direction, thus is written as a function of *θ_k_*. Equation (8) shows that **r̂**_1_ is a noisy weighted sum of the atoms {**ψ**_1_(*θ_k_*) }*_k_*_=1,…_,*_K_*, with each atom corresponding to an incident signal. Therefore, if one can recover the *K* signal components from **r̂**_1_, the signal directions can be determined. In this paper, we first form an overcomplete dictionary **Ψ**_1=_ {**ψ**_1_(*θ*) }*_θ_*_∈ Θ_ on the possible signal direction set **Θ**, and then decompose **r̂**_1_ on **ψ**_1_ under sparsity constraint to obtain the signal components. If the time delays of these multipath signals exist, we should first estimate the time delays following reference [8]. For convenience and more coherent condition, our paper does not consider the time delays.

This sparse decomposition process can be implemented by solving the following convex optimization problem approximately [6,7]:

(9) $$\begin{array}{cc} \min{\left\|\eta \right\|}_{1}, & \text{subject to}{\left\|{\hat{\text{r}}}_{1}-\Psi_{1}\eta \right\|}_{2}\leq \beta , \end{array}$$

where **η** is the energy distribution of **r̂**_1_ on the dictionary, and takes non-zero values of ${\left\{\eta_{\Sigma }^{*}\eta_{k}\right\}}_{k=1}^{K}$ only at the indices of the true signal directions, *β* is the hard threshold of the fitting error between **r̂**_1_ and the observation model **Ψ**_1_**η**, which relies on the perturbation level of **r̂**_1_. The locations of the significant non-zero values in the energy distribution estimate (denoted by **η̂**) indicate the directions of the incident signals. The solution of Equation (9) is single if the number of signals is less than $\left\lfloor \frac{M}{2}\right\rfloor$ [9].

In order to solve Equation (9) for DOA estimation, one should first set *β* according to the perturbation level of **r̂**_1_. The variance of perturbation of **r̂**_1_ can be straightforwardly derived from Equation (6) as:

(10) $$\text{Var}({ɛ}_{1})=\overset{M}{\underset{m=2}{\text{∑}}}\text{Var}\left({ɛ}_{m,1}\right)=\frac{\sigma_{v}^{2}}{N}\overset{K}{\underset{k=1}{\text{∑}}}\overset{K}{\underset{k^{\prime }=1}{\text{∑}}}\eta_{k}\eta_{k^{\prime }}^{*}\left[(M-1)+\overset{M}{\underset{m=2}{\text{∑}}}\psi \left(\tau_{k,m}-\tau_{k^{\prime },m}\right)\right]+\frac{M-1}{N}\sigma_{v}^{4}.$$

Thus the fitting error threshold in Equation (9) can be set according to [10] as:

(11) $$\beta =\mu \times {\left[\frac{\sigma_{v}^{2}}{N}\overset{K}{\underset{k=1}{\text{∑}}}\overset{K}{\underset{k^{\prime }=1}{\text{∑}}}\eta_{k}\eta_{k^{\prime }}^{*}\left((M-1)+\overset{M}{\underset{m=2}{\text{∑}}}\psi \left(\tau_{k,m}-\tau_{k^{\prime },m}\right)\right)+\frac{M-1}{N}\sigma_{v}^{4}\right]}^{\frac{1}{2}}$$

where *μ* is a weighting factor, with an empirical value between 0.5 and 2. To facilitate the calculation of the variance in Equation (10), we rewrite it as follows:

(12) $$\begin{array}{ll} \text{Var}({ɛ}_{1}) & =\frac{\sigma_{v}^{2}}{N}\overset{K}{\underset{k=1}{\text{∑}}}\overset{K}{\underset{k^{\prime }=1}{\text{∑}}}\eta_{k}\eta_{k^{\prime }}^{*}\left[(M-1)+\overset{M}{\underset{m=2}{\text{∑}}}\psi \left(\tau_{k,m}-\tau_{k^{\prime },m}\right)\right]+\frac{M-1}{N}\sigma_{v}^{4}\\ & =\frac{M-1}{N}\sigma_{v}^{2}\left(\overset{K}{\underset{k=1}{\text{∑}}}\overset{K}{\underset{k^{\prime }=1}{\text{∑}}}\eta_{k}\eta_{k^{\prime }}^{*}+\sigma_{v}^{2}\right)+\frac{\sigma_{v}^{2}}{N}\overset{M}{\underset{m=2}{\text{∑}}}\overset{K}{\underset{k=1}{\text{∑}}}\overset{K}{\underset{k^{\prime }=1}{\text{∑}}}\eta_{k}\eta_{k^{\prime }}^{*}\psi \left(\tau_{k,m}-\tau_{k^{\prime },m}\right)\\ & =\frac{M-1}{N}\sigma_{v}^{2}{\text{R}}_{1,1}+\frac{\sigma_{v}^{2}}{N}\overset{M}{\underset{m=2}{\text{∑}}}{\text{R}}_{m,m}. \end{array}$$

In the above expression of Var(ε_1_), **R***_m,m_* and can be approximated by the corresponding elements in **R̂**, $\sigma_{v}^{2}$ and is the unknown noise power. To estimate $\sigma_{v}^{2}$, we first separate the signal and noise subspaces of **R̂** according to the model-order selection techniques, such as MDL [11], then we use the average of the eigenvalues corresponding to the noise subspace to approximate $\sigma_{v}^{2}$. Thus, the fitting error threshold *β* is uniquely determined by the weighting factor *μ*, which relies on the array geometry in use, and can be optimized empirically via sufficient simulations accordingly. In this paper, we choose an 8-element uniform linear array (ULA) for DOA estimation, and *μ* is set to 0.5.

After calculating the fitting error threshold according to Equation (12) and the above approximations, we can solve Equation (9) to reconstruct the signal components from **r̂**_1_, thus estimating the source directions. Various methods can be used for the solution of Equation (9) (see [7] and the references therein), so we do not go deeply into their details, and just turn to the toolbox of SeDuMi [12] for a satisfactory estimate. Finally, the signal directions can be determined according to the locations of the non-zero values in **η̂**. It should be noted that the sparsity constraint is an inner motivation for solving Equation (9), and this constraint helps to concentrate the data energy onto several dictionary atoms corresponding to the signal directions, thus the *a priori* information of signal number is not a necessity for the implementation of the new method. However, the goal of model-order selection is also achieved together with DOA estimation.

## 4. Simulation Results

Suppose two coherent chirp signals impinge onto an 8-element ULA from directions of 10° and 20°, respectively, the central frequency of the two signals is 2.5 MHz with a bandwidth of 40% (the starting and ending frequencies are 2 MHz and 3 MHz accordingly). The initial phases of the two signals are chosen independently and uniformly between 0 and in each trial. The ULA is inter-spaced by half-wavelength of a 2.5 MHz sinusoid, and 512 snapshots are collected at 10 MHz during a chirp period.

As the two signals are completely overlapped in the time-frequency domain, the method in [1] is not able to separate them, thus we choose the conventional coherent signals subspace method (CSM) [13], the iterative method in [2] (denoted as the Wang Method), the focusing method in [3] (denoted as the Gershman Method) and the proposed method to estimate their directions. The central frequency and chirp rate of the incident signals are assumed to be known exactly, the snapshots are divided into sections of 64 snapshots each in CSM, and the iteration number is set to 3 in the Wang Method. The *a priori* signal number information is not used in the proposed method, but is used in the other three methods. As too dense a dictionary may cause an increased estimation bias in the sparse representation techniques [14], we divide the [−90°, 90° ] space into 1° intervals and set the angular samples on the grids to form the dictionary (the searching grid is set identically in the other three methods). If such spatial sampling does not provide the required precision, further grid refinement process [6] or the ML method [4] can be introduced to improve it. The selected model order and coarse DOA estimates can be used to restrict the parameter scope in those further processes to save computational load. In this paper, we skip over those further processes, and concentrate on the performance of adaptability and superresolution in demanding scenarios.

**Figure 1.**
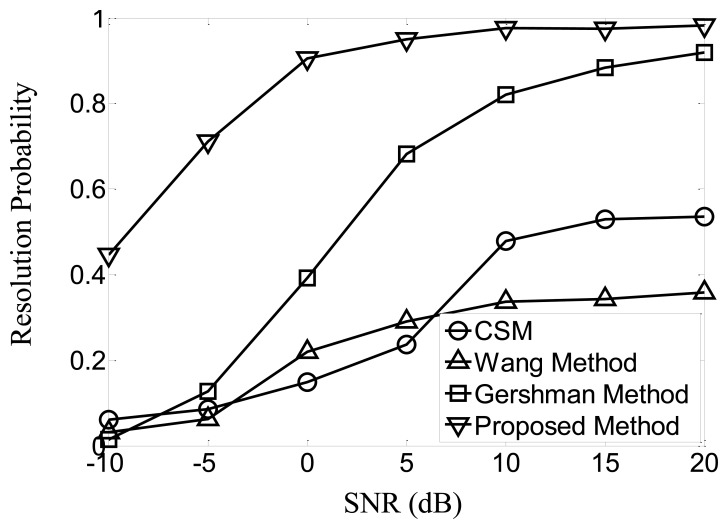
Resolution probabilities at varying SNR.

Firstly, suppose that the SNR of both signals is identical and varies from −10 dB to 20 dB, 1,000 trials are carried out at each SNR. Successful resolution is defined when the two most significant spectrum peaks are located near the true signal directions, and the biases are no larger than 3°. The resolution probabilities of the four methods at various SNR are given in Figure 1. The results indicate that, the proposed method greatly surpasses the other three methods in the given scenarios, and Gershman Method obtains the second best performance, while CSM and the Wang Method fail to achieve satisfying resolution probability, even when the SNR is as high as 20 dB.

**Figure 2.**
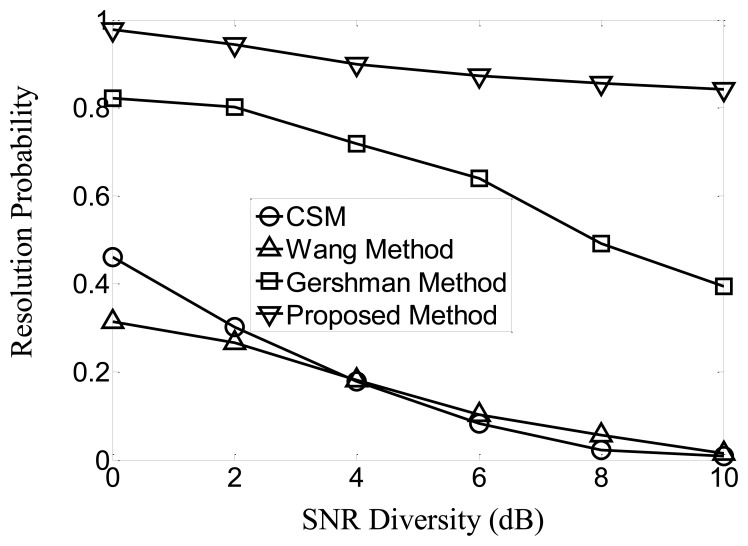
Resolution probabilities at varying SNR diversity.

Then we fix the SNR of the first signal at 10 dB, and attenuate that of the second signal from 10 dB to 0 dB (*i.e.*, the SNR diversity increases from 0 dB to 10 dB) to simulate more vividly the multi-path and echo scenarios. The resolution probabilities of the four methods derived from 1,000 trials are given in Figure 2. The results show that the proposed method is less significantly influenced by the power diversity. It still retains a higher than 80% resolution probability when the SNR diversity is as large as 10 dB. Contrarily, the resolution probabilities of CSM and the Wang Method decrease to 0, and that of the Gershman Method decreases to about 40%.

## 6. Conclusions

The technique of sparse representation to estimate the directions of simultaneous wideband coherent chirp signals is introduced in this paper. The covariance matrix, instead of the time-frequency distribution, is exploited in the new method, and the *a priori* information of signal number is no longer a necessity. Simulation results show that the proposed method greatly surpasses its existing counterparts, especially when the SNR is low or the incident signals are very diverse in amplitude. Moreover, it simultaneously achieves model-order selection.
